# Spatial distribution of neurons innervated by chandelier cells

**DOI:** 10.1007/s00429-014-0828-3

**Published:** 2014-07-24

**Authors:** Lidia Blazquez-Llorca, Alan Woodruff, Melis Inan, Stewart A. Anderson, Rafael Yuste, Javier DeFelipe, Angel Merchan-Perez

**Affiliations:** 1 Laboratorio Cajal de Circuitos Corticales (CTB), Universidad Politécnica de Madrid, Madrid, Spain; 2 Instituto Cajal, CSIC, Madrid, Spain; 3 Department of Biological Sciences, Columbia University, New York, NY USA; 4 Brain and Mind Research Institute, Weill Cornell Medical College, New York, NY 10065 USA; 5 Department of Psychiatry, Children’s Hospital of Philadelphia and University of Pennsylvania School of Medicine, Philadelphia, PA 19104 USA; 6 DATSI, Universidad Politécnica de Madrid, Madrid, Spain

**Keywords:** Chandelier cell, Axo-axonic cell, Pyramidal cell, Axon initial segment, GABA, Three-dimensional reconstruction

## Abstract

Chandelier (or axo-axonic) cells are a distinct group of GABAergic interneurons that innervate the axon initial segments of pyramidal cells and are thus thought to have an important role in controlling the activity of cortical circuits. To examine the circuit connectivity of chandelier cells (ChCs), we made use of a genetic targeting strategy to label neocortical ChCs in upper layers of juvenile mouse neocortex. We filled individual ChCs with biocytin in living brain slices and reconstructed their axonal arbors from serial semi-thin sections. We also reconstructed the cell somata of pyramidal neurons that were located inside the ChC axonal trees and determined the percentage of pyramidal neurons whose axon initial segments were innervated by ChC terminals. We found that the total percentage of pyramidal neurons that were innervated by a single labeled ChC was 18–22 %. Sholl analysis showed that this percentage peaked at 22–35 % for distances between 30 and 60 µm from the ChC soma, decreasing to lower percentages with increasing distances. We also studied the three-dimensional spatial distribution of the innervated neurons inside the ChC axonal arbor using spatial statistical analysis tools. We found that innervated pyramidal neurons are not distributed at random, but show a clustered distribution, with pockets where almost all cells are innervated and other regions within the ChC axonal tree that receive little or no innervation. Thus, individual ChCs may exert a strong, widespread influence on their local pyramidal neighbors in a spatially heterogeneous fashion.

## Introduction

The GABA (γ-aminobutyric acid)-ergic interneurons of the cerebral cortex are a diverse population of cells. Their diversity is manifested in every aspect of their phenotype, as evidenced by their many different morphological, electrophysiological and neurochemical features. Different subtypes of cortical interneurons target different subcellular compartments of the postsynaptic neurons such that there are neurons that innervate only the axon initial segment (AIS), whereas others innervate mainly dendrites or both dendrites and somata with different degrees of selectivity (Ascoli et al. 2008).

Moreover, it has been suggested that in general GABAergic interneurons are not selective for a particular type of neuron (Sohya et al. 2007; Niell and Stryker 2008; Liu et al. 2009) with spatial proximity being the predictor of their connectivity (Bock et al. 2011). A similar conclusion has been reached from examination of connections with those pyramidal neurons located within the axonal arbors of certain subpopulations of GABAergic cells, such as somatostatin-positive (Fino and Yuste 2011; Packer et al. 2013) and parvalbumin-positive neurons (Packer and Yuste 2011; Packer et al. 2013). However, the connections between GABAergic interneurons seem to be more selective. For example, it has been shown that the three major, molecularly distinct interneuron populations—namely, parvalbumin-, somatostatin- and vasoactive intestinal peptide-expressing interneurons—clearly differ in terms of the connections between one another (Pfeffer et al. 2013): parvalbumin-expressing interneurons mainly inhibit one another, whereas somatostatin-expressing interneurons inhibit parvalbumin- and vasoactive intestinal peptide-expressing interneurons and apparently do not inhibit one another. Meanwhile, vasoactive intestinal peptide-expressing interneurons preferentially inhibit somatostatin-expressing interneurons.

Nevertheless, there are many different subtypes of inhibitory cells with widely different anatomical and physiological properties and connectivity patterns (Ascoli et al. 2008) and it is therefore possible that distinct subtypes of cortical GABAergic interneurons may differ in their selectivity for their targets. Thus, identifying classes and subclasses of interneurons is an important step towards understanding how inhibition shapes cortical function (Ascoli et al. 2008; Kepecs and Fishell 2014).

Chandelier cells (ChCs), also known as axo-axonic cells, are among the most distinctive of the GABAergic interneuron subtypes (reviewed in Somogyi et al. 1982, 1998; DeFelipe and Fariñas 1992). ChCs originate in the ventral part of the medial ganglionic eminence (MGE) (Inan et al. 2012) and later in the proliferative zone of the most ventral-lateral region of the lateral ventricle, and migrate through clearly defined routes to achieve a specific laminar distribution in the cortex (Taniguchi et al. 2013). The ChC is a type of “fast-spiking” interneuron, which generally expresses parvalbumin (Ascoli et al. 2008), although some evidence suggests that a certain proportion of them do not (Fish et al. 2013; Taniguchi et al. 2013). These interneurons can be distinguished from other interneurons by the terminal portions of its axon, which form vertical rows of boutons (Ch terminals) resembling candlesticks (Szentagothai and Arbib 1974; Jones 1975). These groups of terminal boutons, or cartridges, target the AIS of pyramidal neurons, forming symmetric synapses (e.g., Somogyi 1977; Fairen and Valverde 1980; Peters et al. 1982; Somogyi et al. 1982; Freund et al. 1983; DeFelipe et al. 1985). Since these synapses are strategically placed where action potentials are generated, they are thought to regulate the generation and back propagation of action potentials, and because a single ChC contacts many pyramidal neurons, they are believed to participate in complex activities such as the synchronization of firing patterns in large populations of pyramidal cells in different functional states (see Klausberger et al. 2003; Howard et al. 2005). Importantly, ChCs have also been implicated in schizophrenia and epilepsy (reviewed in DeFelipe 1999; Howard et al. 2005; Inan and Anderson 2014).

Although ChCs are relatively scarce, several studies have addressed their physiological properties in both the hippocampus and neocortex (Buhl et al. 1994; Klausberger et al. 2003; Tamas and Szabadics 2004; Szabadics et al. 2006; Xu and Callaway 2009; Zaitsev et al. 2009; Glickfeld et al. 2009; Woodruff et al. 2009, 2011). The distribution of cartridges in different areas of the cortex has been reported using presynaptic markers expressed in ChC axon terminals (Inda et al. 2007, 2009), but data concerning the quantitative analysis and spatial distribution of the cartridges of individual ChCs are still scarce. Indeed, only a few cells have been examined. In addition, due to technical difficulties (e.g., incomplete labeling with the Golgi method, the difficulty of 3D reconstruction of the ChC axon coupled with the identification of the postsynaptic target, etc.), meaningful quantitative data are hard to obtain (DeFelipe et al. 1985; Somogyi et al. 1985; Li et al. 1992; Lund and Lewis 1993; Martinez et al. 1996; Krimer and Goldman-Rakic 2001). As a consequence of all of these factors, the spatial connectivity of ChCs is still poorly understood.

Recently, a mouse transgenic line in which ChCs are labeled with green fluorescent protein (GFP) (Woodruff et al. 2009; Inan et al. 2013) has been developed, representing an excellent tool to examine in detail the connectivity of ChCs with pyramidal neurons. Using immunohistochemical detection of axon initial segments in these transgenic mice, it was observed that ChCs innervate neighboring pyramidal neurons in a dense and overlapping manner—a connectivity pattern that may enable ChCs to exert a widespread influence on their local circuits (Inan et al. 2013).

In the present study, we performed whole-cell patch clamp recordings of GFP-labeled ChCs in brain slices of this transgenic mouse line to further examine ChC-pyramidal cell connectivity. Identified GFP-expressing ChCs were intracellularly filled with biocytin and three-dimensional reconstructions of their axons and targets were carried out. In this way we were able to analyze—within individual ChC axonal arbors—the 3D spatial distribution of those neurons innervated and not innervated by Ch terminals.

## Materials and methods

### Animals

We used Nkx2.1-Cre::MADM transgenic mice (P18-23) that express GFP in a subset of neocortical interneurons, including ChCs located in upper cortical layers, most frequently at the border between layers I and II. For the generation of this transgenic line, see Woodruff et al. (2009). Nkx2.1 is a homeodomain transcription factor selectively expressed in the MGE and preoptic area in mid-gestation, and this expression domain becomes restricted to the proliferative zone of the ventral-lateral region of the lateral ventricle, and to a variety of cells in the developing basal ganglia (Sussel et al. 1999; Marin et al. 2000; Xu et al. 2008). Expression of Nkx2.1 is necessary for progenitors to differentiate into parvalbumin-expressing cortical interneurons (Xu et al. 2004) including ChCs (Taniguchi et al. 2013). Animal handling and experimentation were done according to NIH, local IACUC and CSIC guidelines.

### Slice preparation and whole-cell patch clamp recordings

Nkx2.1-Cre::MADM mice were quickly decapitated and 300 μm coronal slices were prepared using a Leica VT1200-S vibratome. The cutting solution contained (in mM): 27 NaHCO_3_, 1.5 NaH_2_PO_4_, 222 Sucrose, 2.6 KCl, 3 MgSO_4_, 0.5 CaCl_2_. Slices were incubated for 30 min at 32 °C in an oxygenated (95 % O_2_ and 5 % CO_2_) artificial cerebrospinal fluid (ACSF, pH = 7.4) solution containing (in mM): 126 NaCl, 3 KCl, 3 MgSO_4_, 1 CaCl_2_, 1.1 NaH_2_PO_4_, 26 NaHCO_3_, and 10 Dextrose. Slices were allowed to equilibrate for at least 30 min at room temperature before being transferred to the recording chamber. The ACSF used for the recordings contained (in mM): 126 NaCl, 3 KCl, 1.5 MgSO_4_, 2.5 CaCl_2_, 1.1 NaH_2_PO_4_, 26 NaHCO_3_, and 10 Dextrose. Whole-cell pipettes contained a solution with (in mM): 135 K-methylsulfate, 8 NaCl, 10 HEPES, 2 MgATP, 0.3 NaGTP, 7 Phosphocreatine, adjusted to pH 7.3 with 1 M KOH. Identity of ChCs was confirmed by their intrinsic firing properties (Woodruff et al. 2009).

### Reconstruction of axonal arbor of ChCs from serial semi-thin plastic sections

**Fig. 1:**
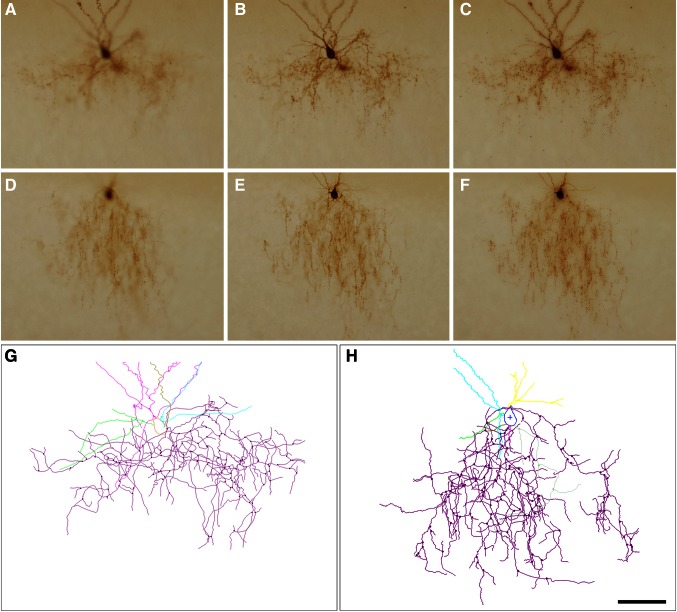
Photomicrographs of two biocytin-injected chandelier cells in 300-µm thick slices. Photomicrographs shown in **a**, **b** and **d**, **e** were taken at different focal planes of the axonal and dendritic arbors of c80520 (ChC1) and b80521 (ChC3) chandelier cells, respectively. **c** and **f** are composite projections comprising 6–8 photomicrographs at different focal planes corresponding to the same ChCs. **g** and **h** represent Neurolucida reconstructions of ChC1 and ChC3, respectively. *Scale bar* (in **h**), 100 µm for **a**–**f**; 70 µm for **g** and **h**

**Fig. 2:**
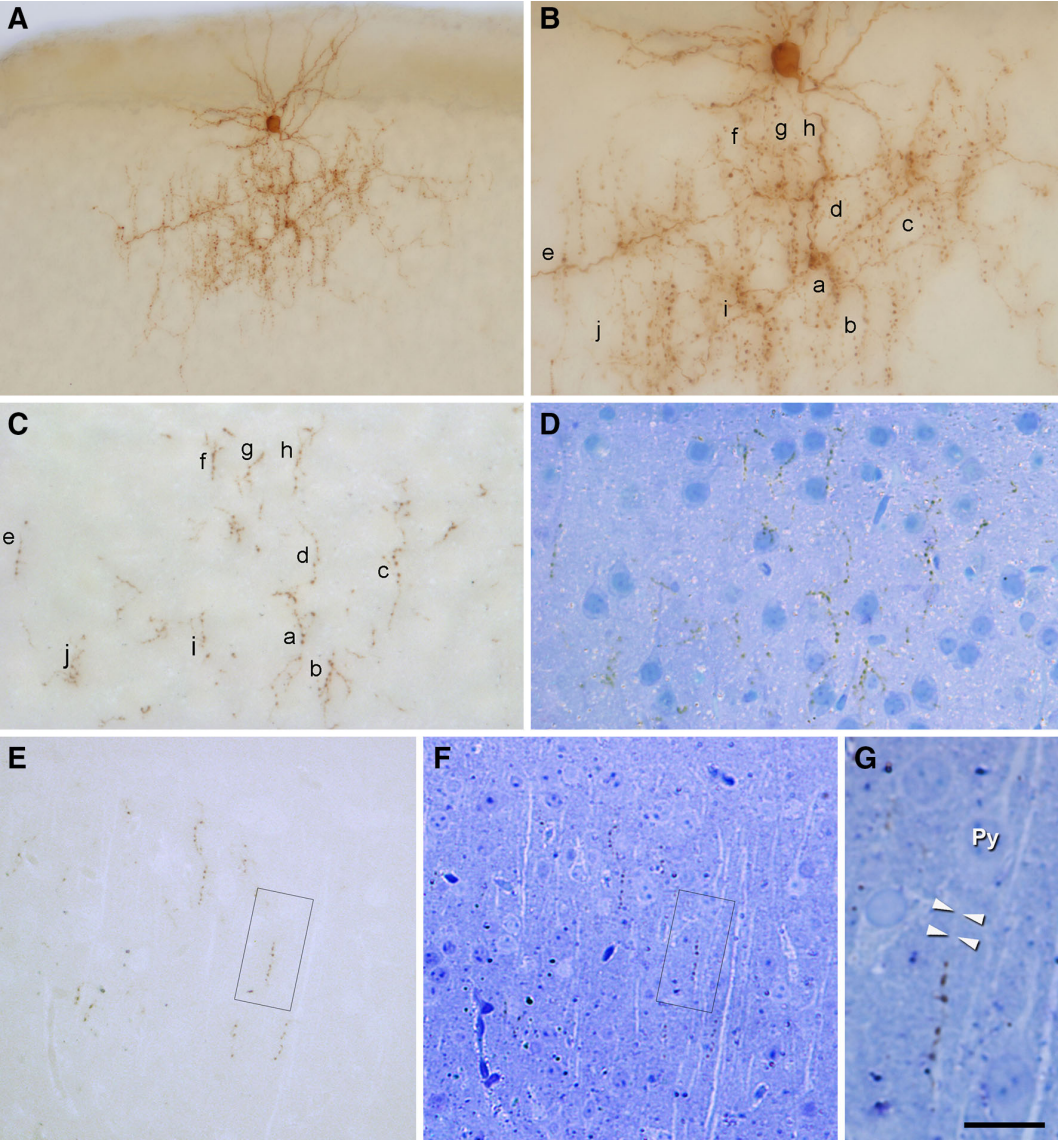
Serial reconstruction of chandelier cells in semi-thin sections. **a** Photomicrograph of a biocytin-filled layer II ChC from a 300 µm thick section embedded in Araldite. **b** Higher magnification of the ChC to illustrate some of the cartridges (*a*–*i*). This 300 µm slice was serially cut into semi-thin (2 µm thick) sections that were photographed (**c**) and then stained with toluidine blue and imaged again (**d**). All cartridges visualized in **a** were identified in the semi-thin sections and photographed. **e**, **f** Semi-thin (1 µm thick) section cut from a different ChC that was imaged before toluidine blue staining (**e**) and after toluidine blue staining (**f**). **g** Details of the area within the *inset* in **e**, **f** showing a biocytin-labeled cartridge opposing the AIS (*arrow heads*) of a pyramidal neuron (Py). *Scale bar* (in **g**), **a** 60 µm; **b**–**d** 35 µm; **e**, **f** 45 µm; **g** 14 µm

A total number of 18 cells from 18 animals were filled with biocytin during whole-cell patch clamp recordings. Slices were then fixed in 4 % paraformaldehyde in 0.1 M phosphate buffer (PB). As previously described, slices were then processed using an avidin–biotin–peroxidase complex, stained with 3,3′-Diaminobenzidine (see Woodruff et al. 2011), imaged with light microscopy before further processing (Fig. 1) and reconstructed with Neurolucida software (MBF Bioscience, Williston, VT, USA). The slices were then post-fixed in 2 % glutaraldehyde in PB for 1 h, treated with 1 % osmium tetroxide in PB for 40 min, dehydrated and flat embedded in Araldite resin. Plastic-embedded sections were serially cut into semi-thin (1–2 μm thick) sections with a Leica EM UC6 ultramicrotome. All but three cells, however, had to be discarded because of incomplete or weak filling, infiltration problems of the resin in the tissue (which is not an infrequent event in patched sections) or because not all serial semi-thin sections could be recovered. The semi-thin sections from the three reconstructed cells were carefully studied under the light microscope and all sections containing the axonal ChC arbor were selected and photographed using a 40× objective. These selected sections were then stained with 1 % toluidine blue in 1 % borax to visualize the neurons and the same fields were imaged again (Fig. 2). The three selected cells were located in layer II/III of the primary somatosensory cortex. Cell c80520 (ChC1) was located in the forelimb region while cells a80519 (ChC2) and b80521 (ChC3) were located in the hindlimb region.

**Fig. 3:**
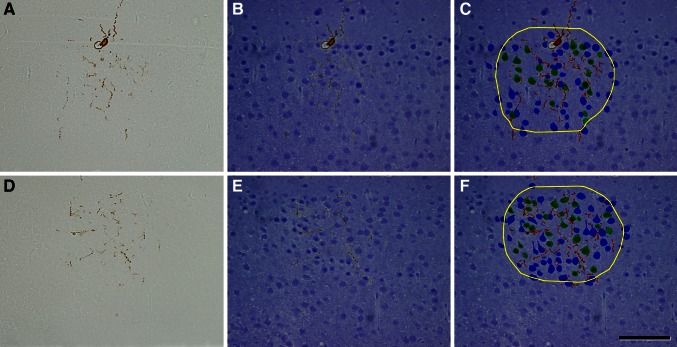
Reconstruction of biocytin-injected chandelier cells and the pyramidal neurons inside their axonal arborizations. Two semi-thin sections of ChC2 before (**a**, **d**) and after staining with toluidine blue (**b**, **e**). **c**, **f** Same semi-thin sections as in **b** and **e**, respectively, with the chandelier soma and processes colored in *red*, the pyramidal cells innervated by a chandelier cell cartridge colored in *green* and the remaining (non-innervated) cells *inside* the axonal arbor of the chandelier cell colored in *blue*. The *border* of the axonal arbor of the chandelier cell in each semi-thin section is indicated in *yellow*. *Scale bar* (in **f**), **a**–**f** 90 µm

**Fig. 4:**
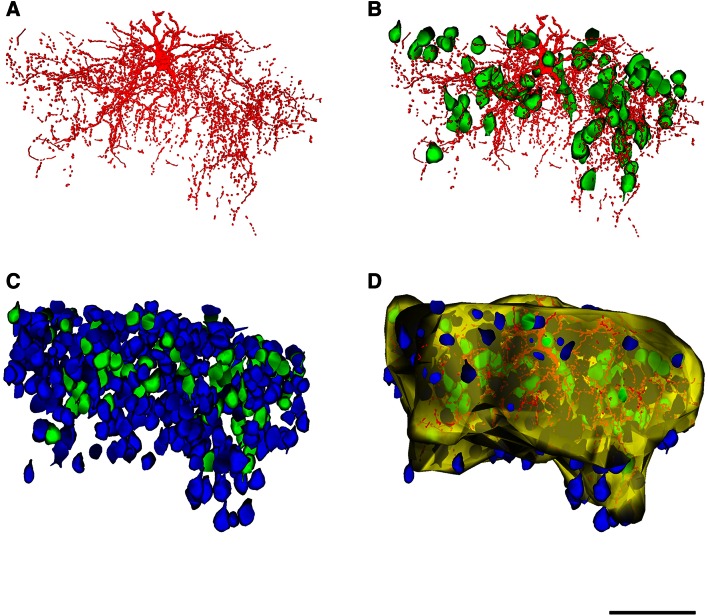
Reconstruction of the chandelier cell c80520 (ChC1). **a** Reconstruction of the soma and processes of the ChC. **b** Same as in **a** but including the neurons (*green*) whose AIS is putatively innervated by the chandelier cell cartridges. **c** All cells inside the axonal arbor of the chandelier cell are shown, including the innervated (*green*) and non-innervated cells (*blue*). **d** Reconstruction of the chandelier cell axonal field and all neurons inside or touching borders of this field in each semi-thin section. The envelope of the chandelier axonal field is represented in *yellow* (see Fig. 3). *Scale bar* (in **f**), **a**–**f** 100 µm

**Fig. 5:**
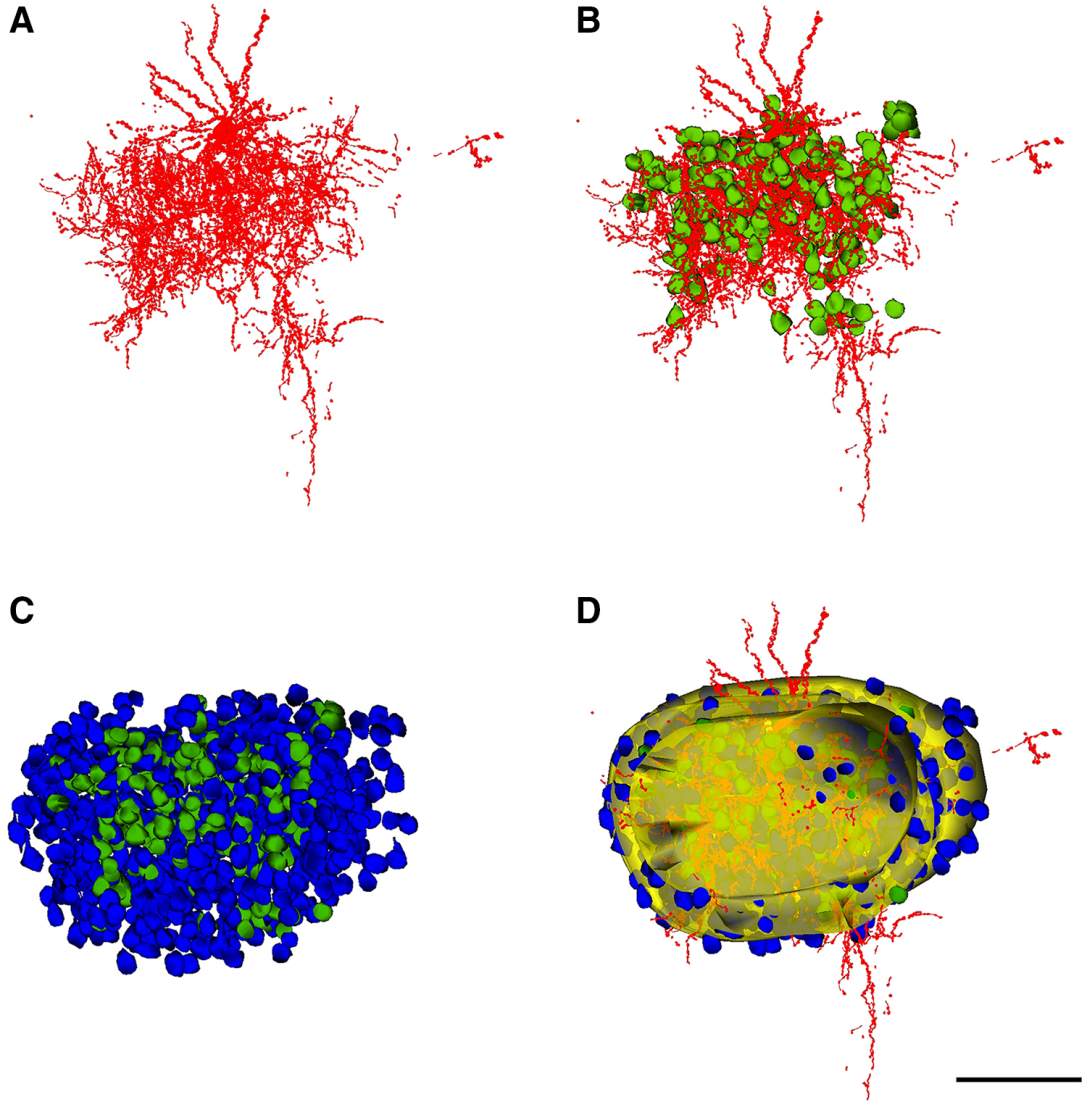
Reconstruction of the chandelier cell a80519 (ChC2). Figure legend: as in Fig. 4. *Scale bar* 100 µm

**Fig. 6:**
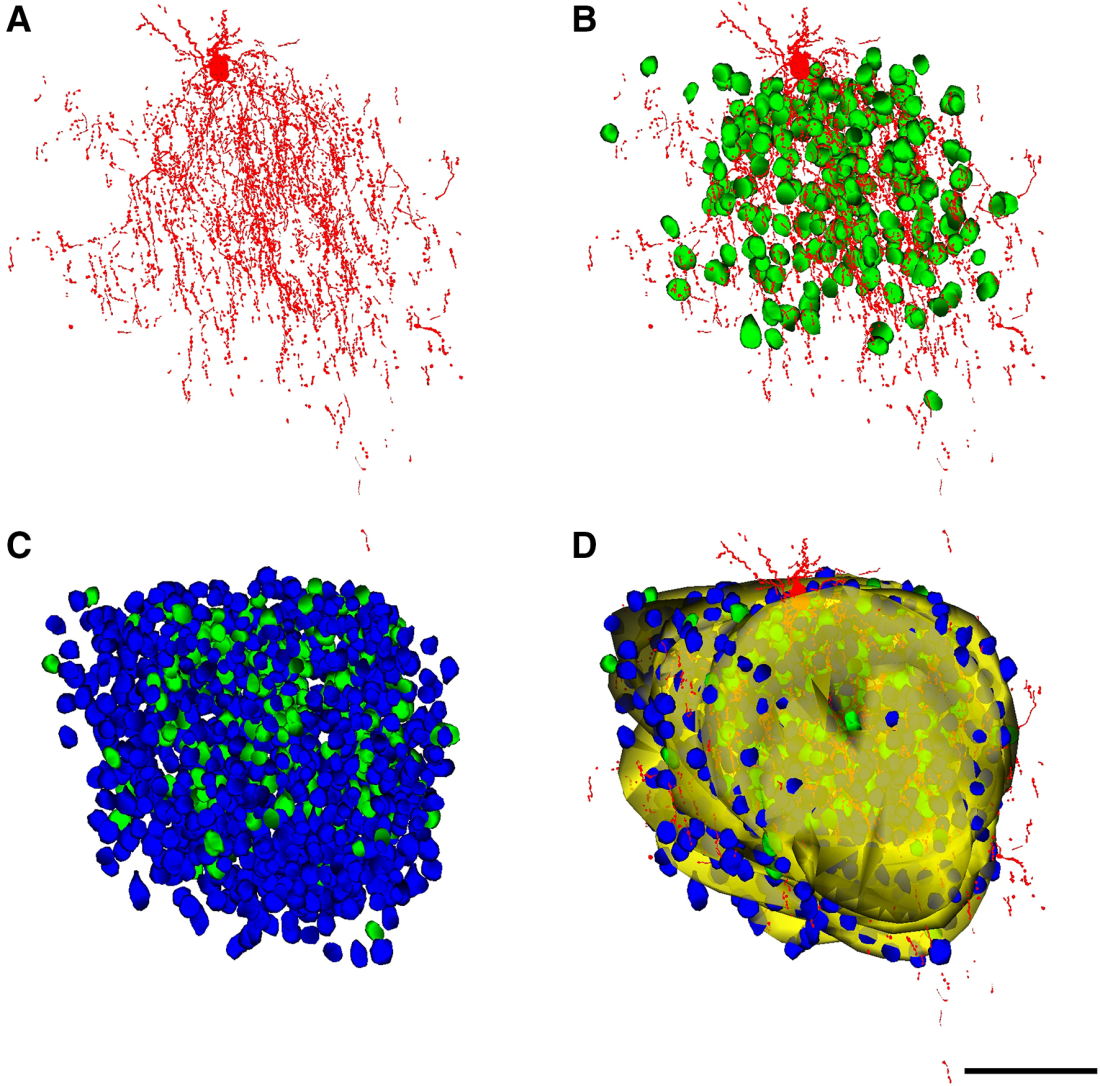
Reconstruction of the chandelier cell b80521 (ChC3). Figure legend: as in Fig. 4. *Scale bar* 100 µm

For ChC1, 28 serial sections of 2 µm were obtained, while for ChC2 and ChC3, 44 and 58 serial sections of 1 µm were used, respectively. Reconstruct Software 1.1.0.0 (Fiala 2005) was used to manually align the images and to carry out the serial reconstruction of ChCs (Fig. 3). The ChC (soma, axonal and dendritic arbor) was pseudocolored in red. To estimate the three-dimensional extent of the ChC axonal arborization, we surrounded with a yellow trace all axonal branches appearing in each semi-thin section (Figs. 3, 4, 5, 6). However, some isolated branches of the periphery of the main axonal arbor were excluded from the analysis (see panels f in Figs. 5, 6). In this way we were able to reconstruct a 3D volume whose shape corresponded to the maximum volume delineated by the distal ends of the main axonal arborization. A neuron was considered to be within the “zone of influence” of the axonal arbor of the ChC if it was inside the axonal tree or if its soma was touching the yellow trace in at least one of the semi-thin sections. Cartridges were identified as vertical rows of two or more boutons opposing the AIS of pyramidal cells. The somata of pyramidal cells whose AIS opposed a cartridge were pseudocolored in green and labeled as Ch+. The somata of pyramidal cells that were inside the axonal arbor (as defined above) but were not innervated by the ChC were pseudocolored in blue and labeled as Ch− (Figs. 3, 4, 5, 6).

### Spatial analysis of the positions of pyramidal cell somata

All reconstructed pyramidal cell somata were exported with Reconstruct software as a vrml file. The three-dimensional position of the centers of gravity or centroids of somata was extracted from the corresponding vrml files with Rhinoceros 4.0 (http://www.rhino3d.com/). Spatial statistical analysis of the position of centroids was performed with SA3D software (Eglen et al. 2008). We used a combination of three commonly used functions (G, F and K functions) to analyze the spatial distribution of Ch+ and Ch− somata (Baddeley et al. 1993; Gaetan and Guyon 2009; O’Sullivan and Unwin 2002). First, nearest neighbor analysis was carried out for all somata. The distribution of distances from each centroid to its nearest neighbor was analyzed by the G function, also called the nearest-neighbor distance cumulative distribution function. This function is estimated using the distances from each centroid to its nearest neighbor, and plotting the fraction of points in the sample that have their nearest neighbor at a given distance or less. To estimate the F function or empty space function, a regular grid is traced within the three-dimensional bounding box that contains the centroids, the distances between each grid crossing point and its nearest neighboring centroid are measured and the cumulative probability of having the nearest centroid at a given distance or less is plotted. Next, K function or Ripley’s function is estimated as the mean number of points within a sphere of increasing radius centered on each sample point. The estimation of G, F and K functions requires that the points to be analyzed are contained within an orthogonal bounding box. Since our samples of centroids were bounded by an irregular ellipsoidal border, tracing a bounding box that includes all points would lead to large empty spaces at the corners that would greatly alter the calculations (especially for the F and K functions). To avoid these artifacts, we used smaller bounding boxes that discarded some of the most peripheral points, but also avoided empty spaces at the corners. Additional statistical analyses were performed with SPSS (IBM Corp., New York, USA).

## Results

In this study, we aimed to determine the spatial distribution of the postsynaptic targets of ChCs and examine whether this distribution follows specific connectivity rules. We made reconstructions of their axonal arbors using semi-thin sections of individual ChCs previously filled with biocytin in the Nkx2.1-Cre::MADM transgenic mice. In this way, we were able to analyze the spatial profile of the biocytin-labeled ChC cartridges of each ChC with high structural resolution.

### 3D reconstruction of ChCs and the neurons within their axonal arbor

Three ChCs filled with biocytin (ChC1, ChC2 and ChC3) (Fig. 1) were selected and further processed to obtain serial semi-thin sections for carrying out the complete ChC arbor reconstruction. Putative postsynaptic pyramidal neurons were identified by their typical somatic morphologies revealed by counterstaining with toluidine blue. Biocytin-labeled boutons of ChC cartridges were observed to be opposing the AISs arising from pyramidal cell somata (Figs. 2, 3).

**Fig. 7:**
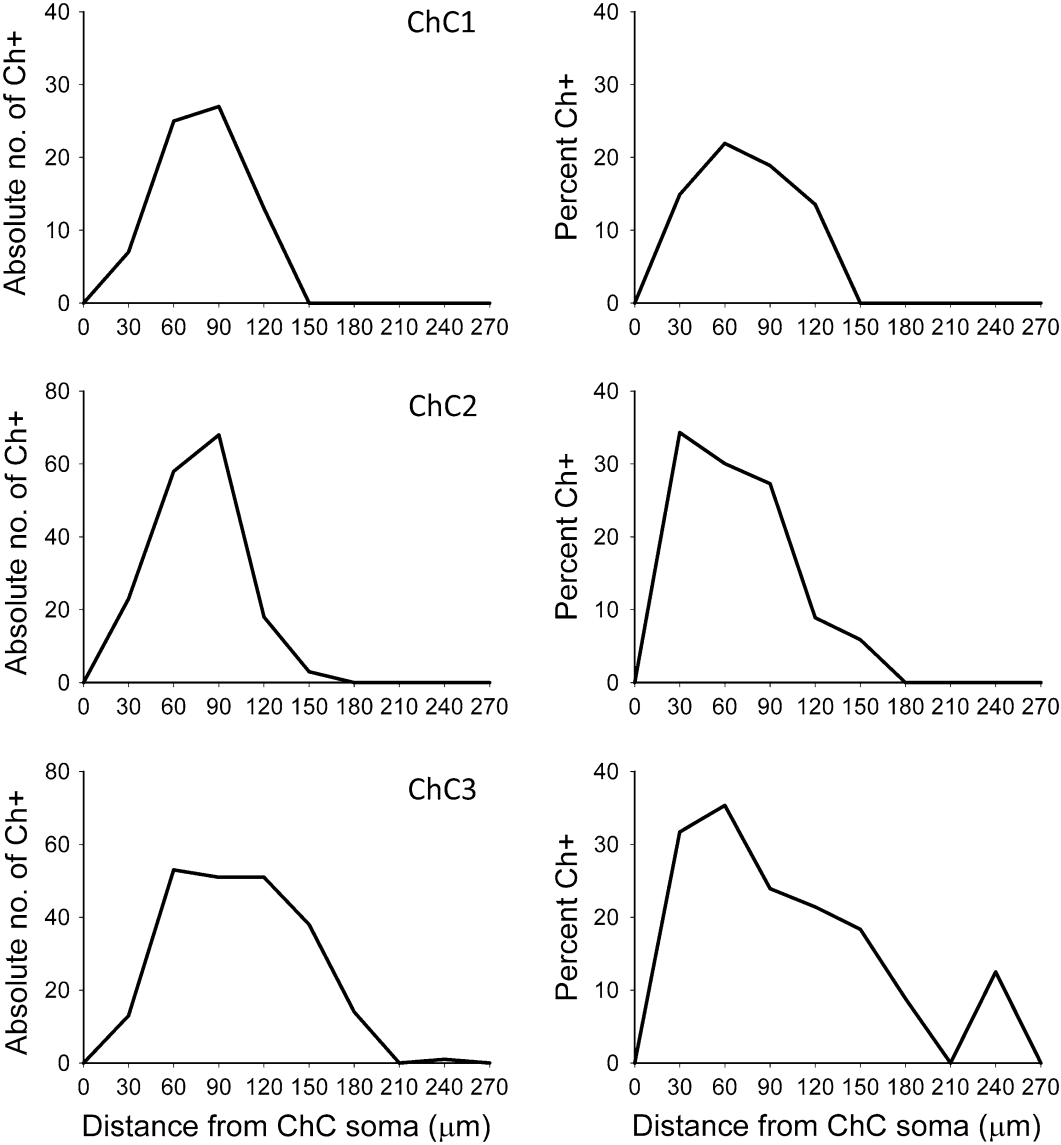
Sholl analysis of pyramidal cells innervated by three reconstructed chandelier cells (ChC1, ChC2, ChC3). The *curves* represent the absolute number (*left column*) and the percentage (*right column*) of innervated pyramidal cells (Ch+) at different distances from the ChC soma

The axonal and dendritic arbors of three ChCs were reconstructed in 3D from serial semi-thin (1–2 μm thick) sections. We determined the extent of cortical territory encompassed by the distal terminations of the main axonal arbor and counted the pyramidal neurons located inside it or touching its borders (see "Materials and methods"). All neuronal cell bodies within the axonal arbor were reconstructed and were scored as innervated (Ch+) when two or more axonal boutons lined up vertically opposing the pyramidal cell AIS (Figs. 4, 5, 6). Non-innervated pyramidal cells (Ch−) within the axonal arbor were also counted. The total numbers of pyramidal cells within the axonal arbor were 405, 762 and 1,081 in ChC1, ChC2 and ChC3, respectively. The absolute numbers (and percentages) of cells that were innervated by the reconstructed axonal trees of ChC1, ChC2 and ChC3 were 72 (17.78 %), 170 (22.31 %) and 221 (20.44 %), respectively. The spatial positions of pyramidal cell somata were represented by their centers of gravity or centroids. We performed a Sholl analysis to determine the distribution of Ch+ cells at different distances from the ChC soma. The analysis showed that the highest numbers of Ch+ cells were preferentially located 30–120 μm from the ChC soma (Fig. 7). When the percentage of Ch+ was considered (instead of absolute numbers), it peaked at 30–60 μm from the ChC cell body (Fig. 7).

**Fig. 8:**
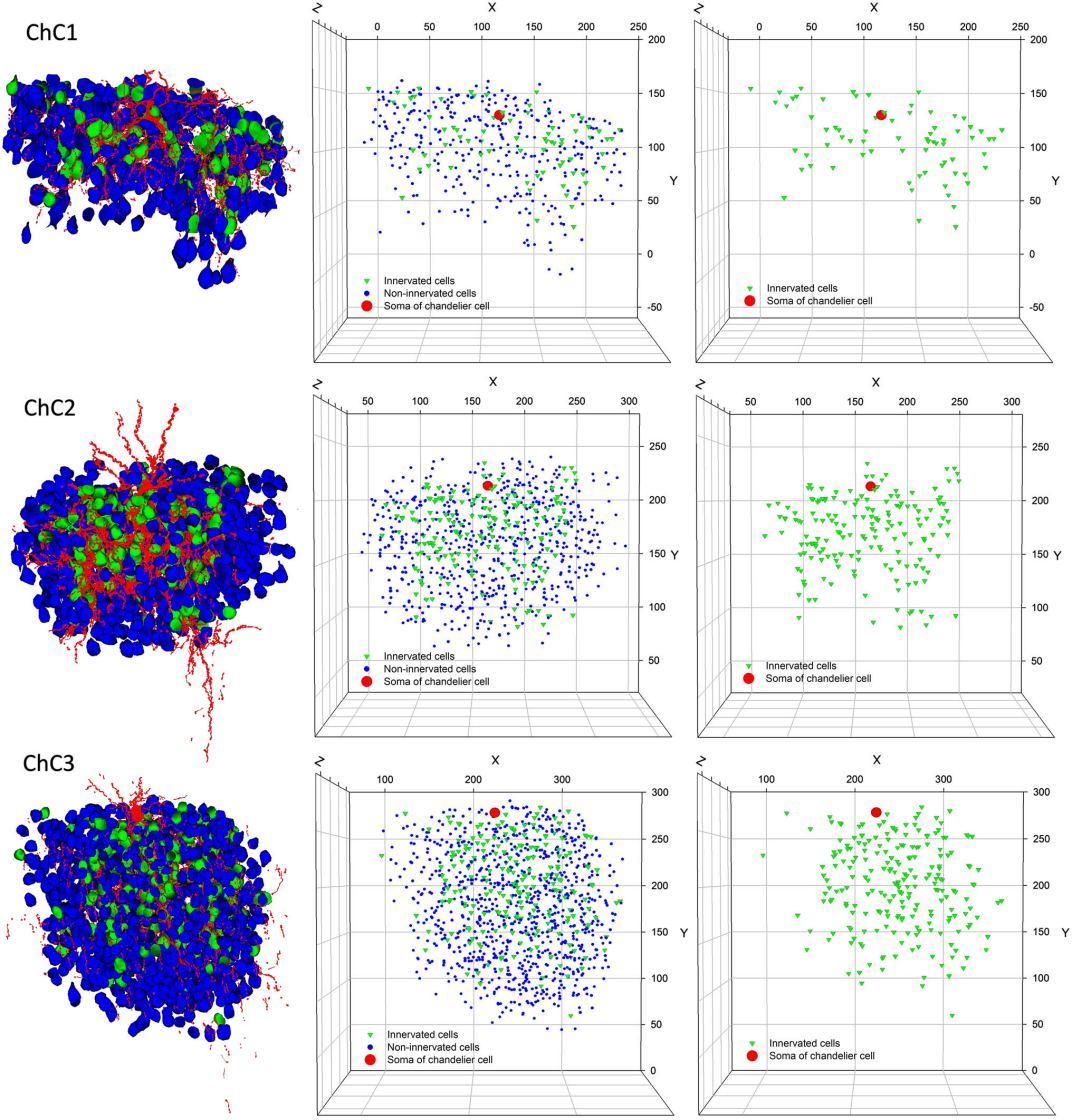
Extraction of pyramidal cell positions within the axonal arbor of three reconstructed chandelier cells (ChC1, ChC2, ChC3). In the *left column*, the soma and processes of three ChCs have been represented in *red*. Pyramidal cells that are innervated by these ChCs have been represented in *green*, while non-innervated cells inside their axonal arbor have been represented in *blue*. In the center column, the spatial positions of the centroids of innervated (*green*) and non-innervated pyramidal cells (*blue*) have been plotted, together with the soma of the corresponding ChC (*red*). In the *right column*, only the centroids of innervated cells (*green*) and the ChC somata (*red*) have been represented. Note that different scales (in µm) have been used in the plots corresponding to the different ChCs

Analysis of the three-dimensional positions of Ch+ pyramidal neurons revealed that the innervation pattern was heterogeneous, with pockets of cortical territory where every neuron seemed to be innervated and other zones, located within the territory covered by the ChC axon seemed to be occupied exclusively by non-innervated neurons (Fig. 8). In order to examine this apparent microheterogeneity more accurately, we calculated the G, F and K functions of the centroids of all cells within the reach of the axonal trees of ChCs.

**Fig. 9:**
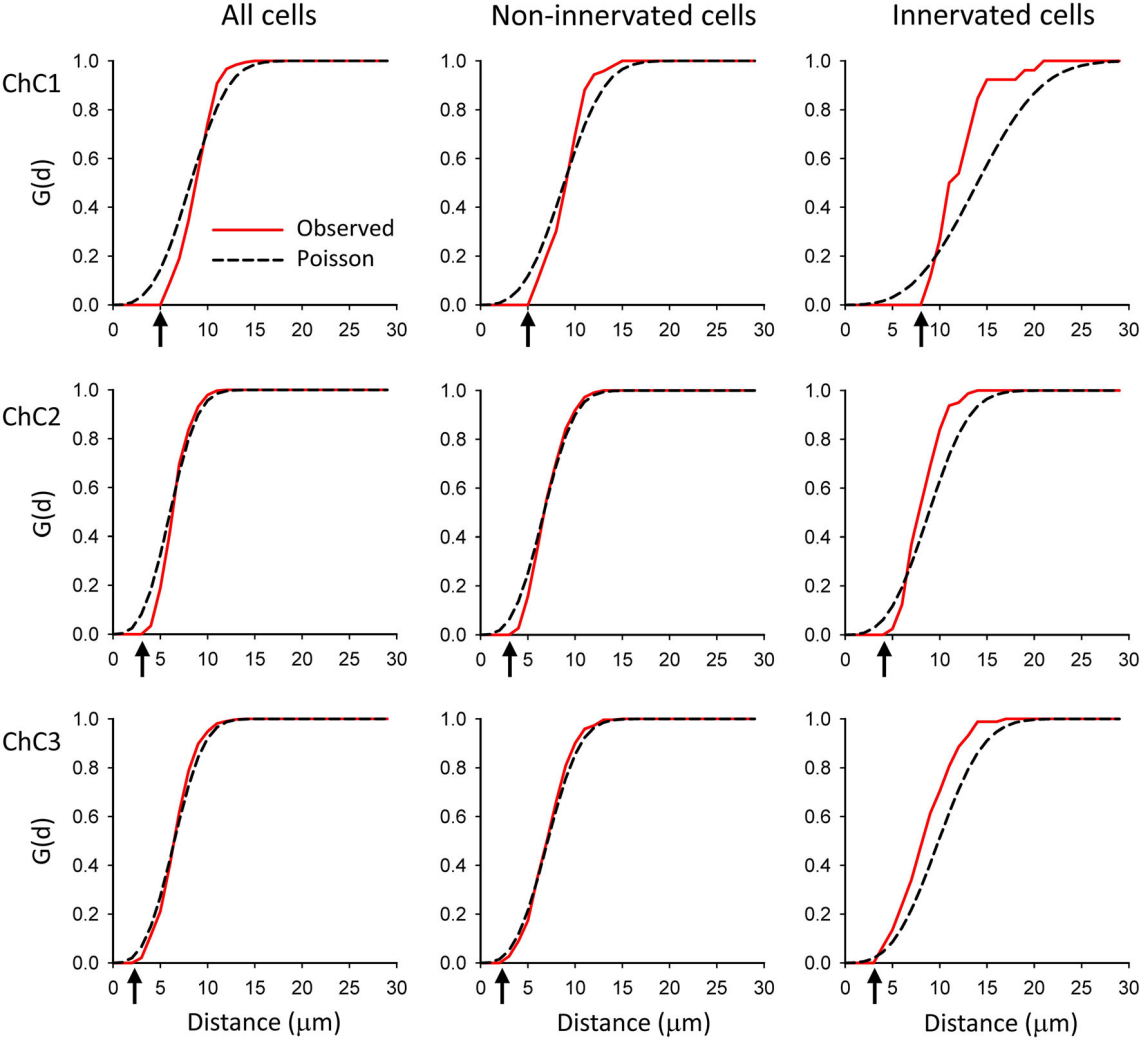
G functions calculated from the distances to the nearest neighbors of pyramidal cells within the axonal arbor of three reconstructed ChCs (ChC1, ChC2, ChC3, *top* to *bottom rows*). The spatial position of the centers of gravity or centroids of cell somata was used for the calculations. G functions represent the fraction of cells that have a nearest neighbor at a given distance or less. The experimentally observed G functions (*red* *continuous* traces) and the G functions corresponding to theoretical homogeneous Poisson processes (*black dashed* traces) have been represented. For each ChC, three groups of cells were studied. First, all pyramidal cells that were located within the axonal tree of the ChC were analyzed (*left column*). Second, only cells that were not innervated by the ChC were included (*mid column*). Third, only cells whose axon initial segment was innervated by a cartridge were considered (*right column*). In all ChCs, all cells inside the axonal tree and non-innervated cells showed G functions that were very similar to those of the corresponding Poisson processes. When only innervated cells were analyzed, observed G functions rose more rapidly than the corresponding Poisson process, indicating that nearest neighbors were closer than would be expected for a homogeneous Poisson process. In all cases the observed G functions showed a dead space at short distances (*arrows*) where the probability of finding a nearest neighbor was zero or very low. This is due to the fact that cells cannot overlap in space, which limits how close their centroids can be to one another

**Fig. 10:**
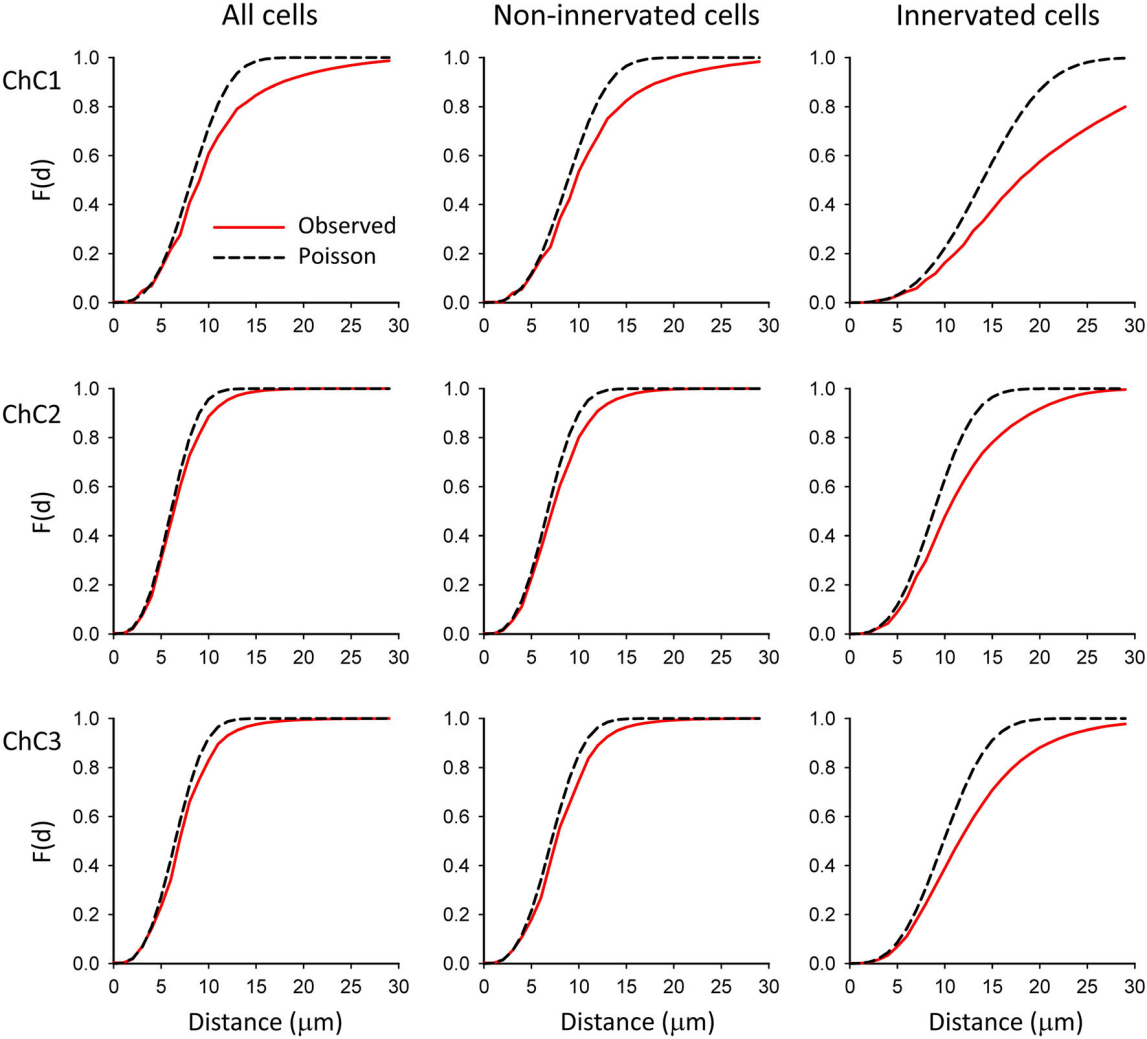
F functions corresponding to the pyramidal cells within the axonal arbor of three reconstructed ChCs (ChC1, ChC2, ChC3, *top* to *bottom rows*). The spatial position of the centers of gravity or centroids of cell somata was used for the calculations. The experimentally observed F functions (*red continuous* traces) and F functions corresponding to theoretical homogeneous Poisson processes (*black dashed* traces) have been represented. All pyramidal cells that were located within the axonal tree of the ChC (*left column*) as well as those cells that were not innervated by the ChC (*mid column*) showed F functions that were very similar to the corresponding Poisson process (ChC2 and ChC3) or were displaced to the *right* (ChC1). In all ChCs, when only innervated cells were analyzed (*right column*) experimental F functions clearly rose more slowly than the theoretical Poisson F functions

**Fig. 11:**
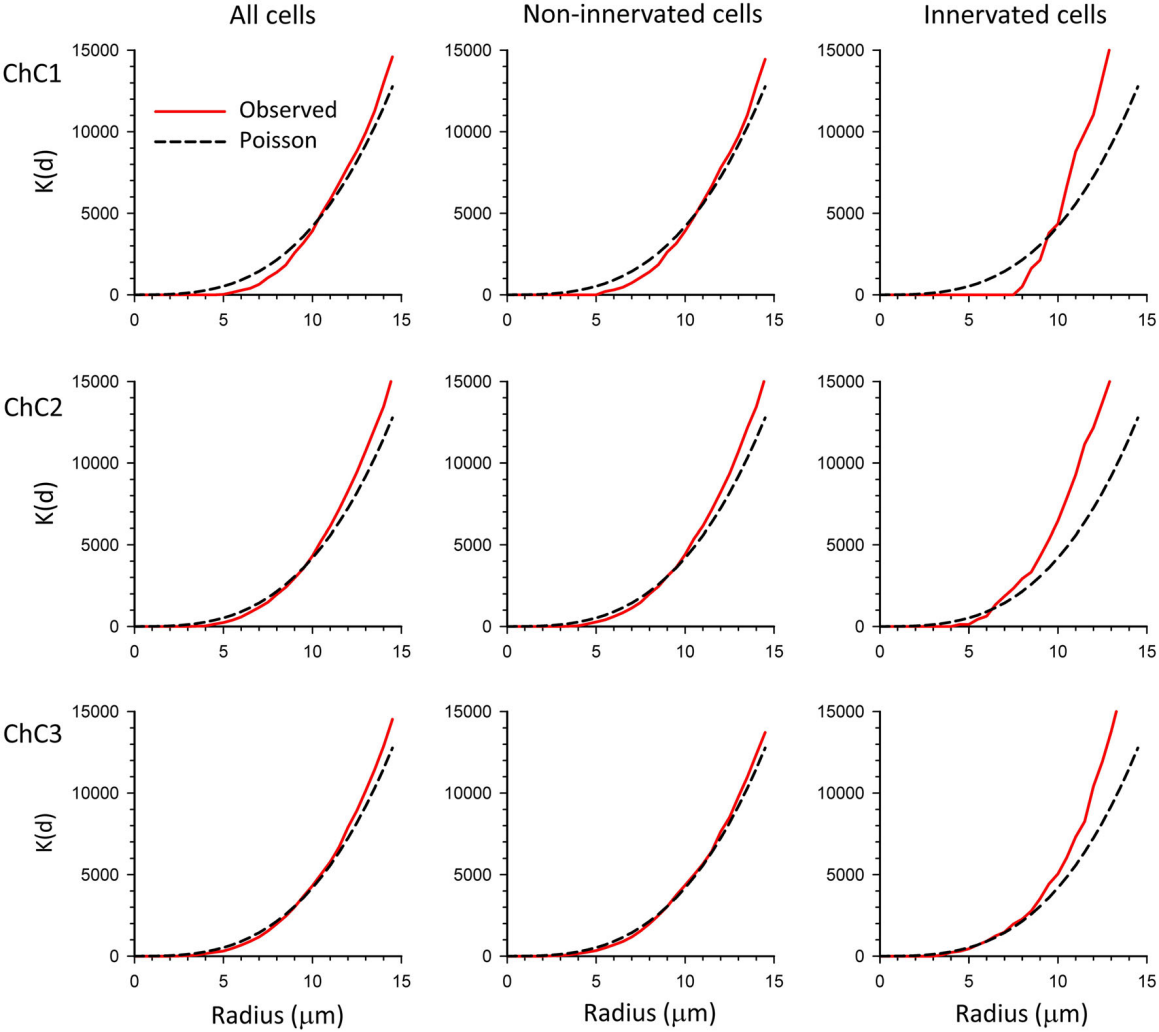
K functions calculated from the spatial positions of neuronal cell somata (represented by their centroids) within the axonal arbor of three reconstructed ChCs (ChC1, ChC2, ChC3). The K functions show the cumulative mean density of centroids within a sphere of increasing radius centered on each sample point. The experimentally observed K functions (*red continuous* traces) and K functions corresponding to a theoretical homogeneous Poisson process (*black dashed* traces) have been represented. In all chandelier neurons, the K functions for all cells (*left column*) and non-innervated cells (*mid column*) were similar to the corresponding Poisson process, except for the fact that they showed a dead space at short distances (see also Fig. 9). Conversely, innervated cells showed K functions that rapidly climbed to higher densities than would be expected in a Poisson process (*right column*)

First, we examined the spatial distribution of the pyramidal cells whose AIS was and was not opposed by a cartridge (Ch+ and Ch−, respectively). The mean nearest-neighbor distances between the centers of gravity or centroids of cell somata (Table 1) revealed no statistically significant difference between Ch+ and Ch− cells in any of the three ChC arbors (Mann–Whitney and two-sample Kolmogorov–Smirnov tests). We then explored whether the three-dimensional positions of pyramidal cells innervated by each ChC could be described by any of the three basic patterns of spatial distributions: complete spatial randomness (CSR), regular or clustered patterns (see Illian et al. 2008; Gaetan and Guyon 2009). In CSR or homogeneous spatial Poisson point process, points are equally likely to occur anywhere in space and the position of each point is independent of any other point in the sample. In a regular or dispersed pattern, the points are located as far as possible from their neighbors, and they tend to form a regular, lattice-like pattern. Finally, in a clustered distribution, the points are concentrated in some regions of space while other regions contain few or no points. Although there are no clear-cut limits between these three basic patterns, CSR represents a boundary condition between clustered and dispersed spatial processes. To analyze the spatial distribution of Ch+, Ch− and all somata, we calculated the G, F and K functions based on the positions of cell somata centroids (Figs. 9, 10, 11, see "Materials and methods"). The theoretical curves corresponding to a homogeneous Poisson process or CSR were also plotted for each graph (black, broken lines in Figs. 9, 10, 11). The G functions (representing the nearest-neighbor distance cumulative distribution) for all somata and for Ch− somata revealed a pattern of distribution that did not greatly depart from a Poisson distribution in the three ChCs studied (Fig. 9). However, the G functions for Ch+ somata (and hence for ChC cartridges) were steeper than expected if somata were distributed at random under CSR conditions, revealing closer than expected nearest neighbors and thus suggesting a clustered pattern (Fig. 9). G function curves also indicated that there is an empty space around all centroids where the probability of having a nearest neighbor is zero or very low. This dead space (indicated with arrows in Fig. 9) is mostly due to the fact that centroids cannot be too close to each other since the volumes they represent, the cell somata, cannot overlap in space.

**Table 1:** Nearest-neighbor distances

	**Type of nearest neighbor**	
	Ch+	Ch−
***ChC1***		
**Type of cell**		
Ch+	9.45 ± 0.91	8.98 ± 1.75
Ch−	9.38 ± 2.26	8.93 ± 1.99
***ChC2***		
**Type of cell**		
Ch+	6.87 ± 1.38	6.47 ± 1.68
Ch−	6.38 ± 1.78	6.77 ± 1.68
***ChC3***		
**Type of cell**		
Ch+	6.97 ± 2.17	6.89 ± 2.12
Ch−	7.01 ± 2.02	7.14 ± 2.06

Mean nearest-neighbor distances ± SD between the centroids of the pyramidal cells inside the axonal tree of three reconstructed ChCs (ChC1, ChC2, ChC3). Pyramidal cells were labeled Ch+ when they received innervation from the ChC and Ch− when they were not innervated. No statistically significant differences were found between the innervated and non-innervated cells within the same axonal arbor. All distances are given in micrometers

Regarding the F functions or empty space functions (Fig. 10), all pyramidal cells that were located within the axonal tree of the ChC and Ch− showed F functions that were very similar to the corresponding Poisson process (ChC2 and ChC3) or were slightly displaced to the right (ChC1). In all ChCs, when only Ch+ were analyzed, experimental F functions clearly rose more slowly than Poisson F functions. This suggests the presence of regions where the number of somata is lower than expected for a random Poisson distribution, as would be the case in a clustered pattern.

K functions were also calculated for Ch+, Ch− and all somata, along with the K functions corresponding to the theoretical CSR or Poisson process (Fig. 11). In this function, the mean number of points within a sphere of increasing radius centered on each sample point is plotted. Similar to the G function plots, K function graphs for the centroids of all somata and Ch− did not greatly depart from the K functions corresponding to a Poisson process. K functions for Ch+ somata showed higher than expected point densities. A dead space around centroids was observed with K functions, similar to the observations mentioned above for G function analysis (Fig. 11).

Taken together, the G, F and K functions suggest a clustered pattern for Ch+ somata. This does not necessarily mean that they are spatially segregated from Ch− somata. If Ch+ and Ch− cells were intermingled at random (no spatial segregation), the probability of having a nearest neighbor of the same or different type would only depend on the proportion of both types of cells in the general population. In the case that Ch+ and Ch− somata are spatially segregated, the probability of having a nearest neighbor of the same type would be higher than expected. To test this, 2 by 2 contingency tables were created showing both types of somata against the type of their nearest neighbor. Fisher’s exact test was applied to these tables indicating that the somata innervated by ChC1 were intermingled at random with non-innervated somata, while cell somata innervated by Ch2 and Ch3 were spatially segregated (Table 2).

**Table 2:** Contingency tables showing the type of pyramidal cell soma against the type of their nearest neighbor within the axonal tree of three reconstructed chandelier cells (ChC1, ChC2, ChC3)

	**Type of nearest neighbor**		
	Ch+	Ch−	Total
***ChC1***			
Fisher’s exact test *p* = 0.8053			
** Type of cell**			
Ch+			
**Observed counts**	**7**	**31**	**38**
*Expected counts*	*6.20*	*31.80*	*38.00*
Ch−			
**Observed counts**	**23**	**123**	**146**
*Expected counts*	*23.80*	*122.20*	*146.00*
Total			
**Observed counts**	**30**	**154**	**184**
*Expected counts*	*30.00*	*154.00*	*184.00*
***ChC2***			
Fisher’s exact test *p* = 0.0140			
** Type of cell**			
Ch+			
**Observed counts**	**45**	**74**	**119**
*Expected counts*	*34.46*	*84.54*	*119.00*
Ch−			
**Observed counts**	**63**	**191**	**254**
*Expected counts*	*73.54*	*180.46*	*254.00*
Total			
**Observed counts**	**108**	**265**	**373**
*Expected counts*	*108.00*	*265.00*	*373.00*
***ChC3***			
Fisher’s exact test *p* = 0.0035			
** Type of cell**			
Ch+			
**Observed** **counts**	**58**	**120**	**178**
*Expected counts*	*42.74*	*135.26*	*178.00*
Ch−			
**Observed counts**	**124**	**456**	**580**
*Expected counts*	*139.26*	*440.74*	*580.00*
Total			
**Observed counts**	**182**	**576**	**758**
*Expected counts*	*182.00*	*576.00*	*758.00*

Pyramidal cells were either innervated (Ch+) or not innervated (Ch−) by cartridges of the corresponding chandelier cell. The observed counts are shown in bold. The expected counts (in italics) are calculated from the marginal totals. Fisher’s exact test was applied to the contingency tables to determine whether the cells were intermingled at random or spatially segregated. The resulting p value is given in the upper left corner of each table. Results showed that Ch+ and Ch− cells inside the axonal arbor of ChC1 were intermingled at random, since the probability of having a nearest neighbor of the same or different type was the expected probability given the proportions of Ch+ and Ch−. However, ChC2 and ChC3 showed spatial segregation, since any given cell (either Ch+ or Ch−) has a higher than expected probability to have a nearest neighbor of the same type

## Discussion

The major findings of the present study are twofold. First, the overall percentage of neurons that were innervated by ChCs within their axonal arbors was around 20 %. Second, the neurons innervated by a ChC follow a clustered distribution, even showing spatial segregation (ChC2 and ChC3), meaning that pockets of very dense ChC innervation exist, whereas other regions remain non-innervated. Thus, we propose that individual ChCs exert a strong, widespread influence on their local neighbor neurons in a spatially heterogeneous manner.

The overall percentage of neurons that were innervated by the three reconstructed ChCs within their axonal arbor was around 20 %, with a peak of 22–35 % at distances of 30–60 µm from the ChC somata, decreasing to lower percentages with increasing distances. These figures must be taken as the lower boundary since the absolute numbers and percentages of innervated cells could have been underestimated due to several factors (see also Inan et al. 2013): (i) our samples were obtained from brain slice preparations, and it is likely that axon collaterals may have been damaged by the slicing such that we are not observing the full extent of the ChC axons; (ii) It is possible that our method does not completely reveal the full extent of the ChC axon because the biocytin filling of the ChC could have been incomplete; (iii) The criterion for the identification of ChC terminal–AIS contact requires at least 2 adjacent boutons to be present; (iv) The definition of the denominator of the percentage equation may include neurons which are located too far from the ChC axon for them to be realistically innervated by the axon in question. This is a particularly relevant issue since ChC axons only make synaptic contacts with AIS, so being even a couple of microns away from the AIS may impede the connection. When taking all these factors into account, it can be concluded that the actual connectivity rates should be higher, and that ChC axons may contact a greater number of their neighboring neurons than estimated in the present study. In fact, we found some territories where every single pyramidal cell was innervated (see below). Moreover, previous results indicate that ChC axonal trees overlap so that a single AIS is innervated by an average of 3.8 different ChCs with each of them contributing an average of 4 boutons per AIS (Inan et al. 2013). In addition, since axonal arbors overlap, it is possible for pyramidal cells that are not innervated by an individual ChC to be innervated by one or several neighboring ChCs. Nevertheless, with the caveats expressed above, if all pyramidal cells located within the axonal arborizations of ChCs were innervated, then ChCs would display a much denser axonal arbor. For example, the total number of pyramidal cells within the axonal arbor was 405, 762 and 1,081 in ChC1, ChC2 and ChC3, respectively, whereas the numbers of cells that were innervated by these cells were 72 (17.78 %), 170 (22.31 %) and 221 (20.44 %), respectively.

**Fig. 12:**
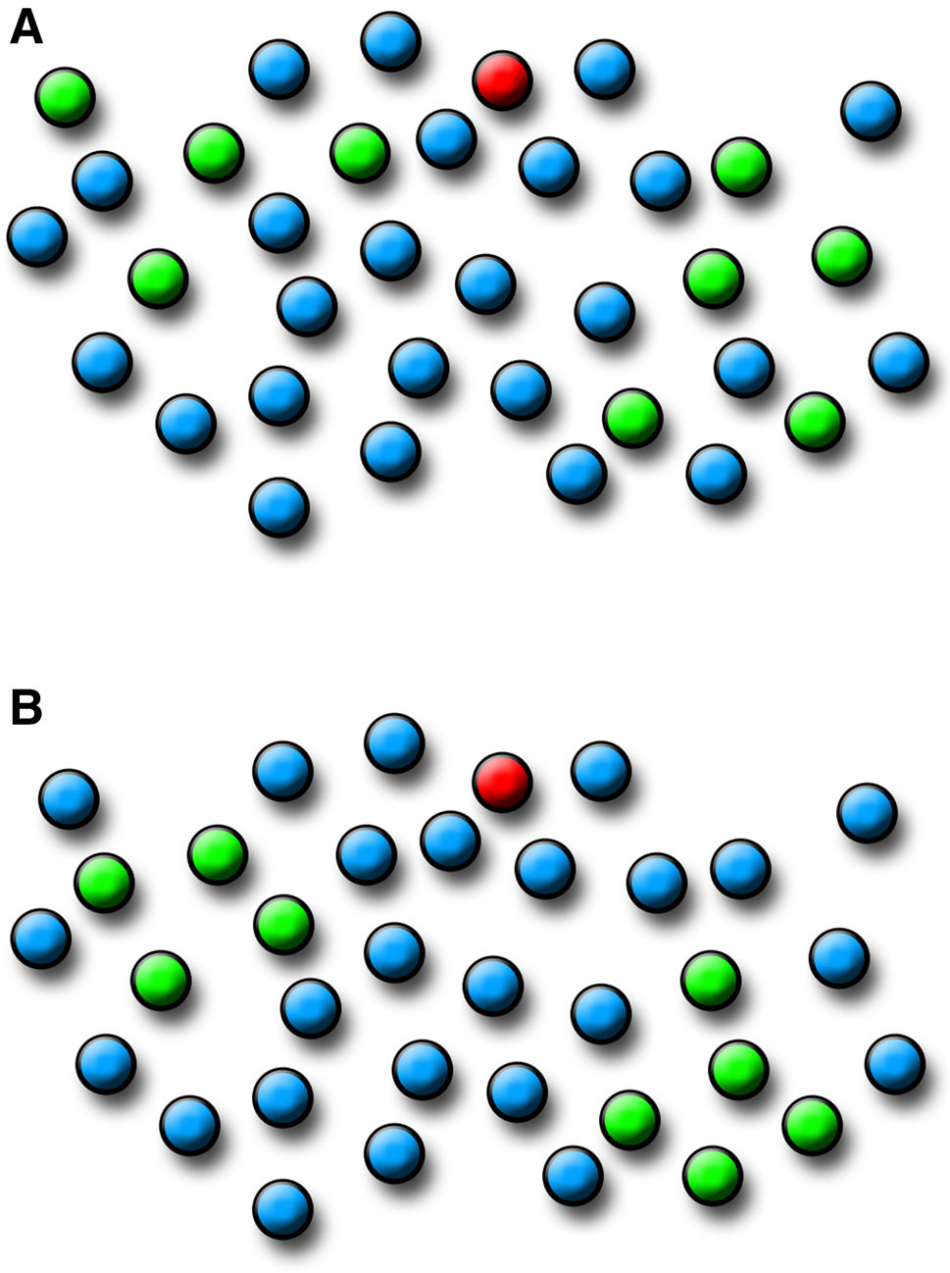
Diagram showing two models for the spatial distribution of pyramidal neurons inside the axonal arbor of ChCs. The soma of the ChCs has been represented in *red*, pyramidal cells whose AIS is innervated by a ChC cartridge are represented in *green* (Ch+) and non-innervated pyramidal cells are shown in *blue* (Ch−). In **a**, innervated cells follow a clustered pattern but are intermingled at random with the predominant non-innervated cells. In **b**, innervated cells also follow a clustered pattern, although in this case they are spatially segregated from non-innervated cells. Note that distances between neurons are the same in both cases

Our results are in line with previous studies showing a widespread innervation of neurons by single ChCs (DeFelipe et al. 1985; Somogyi et al. 1985; Li et al. 1992; Inan et al. 2013; Tai et al. 2014) and we have also extended these findings by providing additional data on the spatial distribution of the terminals (and hence innervated pyramidal cells) of individual ChCs. Since none of the functions F, G and K alone suffice for the characterization of a point pattern, we have used them in combination. It was observed that the neurons innervated by individual ChCs consistently follow a clustered pattern, thus confirming what has been qualitatively observed previously in various species including rat, mouse, cat and monkey (Fairen and Valverde 1980; Somogyi et al. 1982; DeFelipe et al. 1985; Li et al. 1992; Inan et al. 2013). This implies the existence of pockets of dense innervation, as well as other regions where pyramidal cells apparently receive scarce or no innervation from that single ChC. In addition, we have shown that innervated pyramidal cells can intermingle with non-innervated cells or can be spatially segregated from them. Thus, we propose two possible models for the distribution of innervated pyramidal cells inside the axonal tree of a ChC (Fig. 12). In both models, innervated cells show a clustered pattern, but in one of them they are intermingled at random with non-innervated cells (Fig. 12a) while in the other they form clusters comprising mainly innervated cells (Fig. 12b). However, the difference between the two models is subtle and can only be identified by specific statistical tools. At present, we do not have enough information to decide whether these two models actually correspond to different patterns of innervation, or whether they have any relationship with synaptic specificity.

The underlying mechanism or mechanisms that give rise to this clustered distribution remain unclear. It is possible that it is simply due to the abundance or lack of axonal branching in different regions or, alternatively, it could be due to a specific preference of the axon for certain neuronal groups over others, as if there may be pockets of pyramidal neurons that are actively selected or avoided by the ChC axons. Unfortunately, both possibilities will generate a non-random spatial distribution of innervated cells, so we cannot yet discern them. At the same time, there is evidence in the literature for differences in innervation by ChCs of different pyramidal targets. For example, in the cat visual cortex, the number of symmetric synapses on the AIS of cortico-thalamic projecting pyramidal neurons is extremely low (from 1 to 5 per neuron) compared to callosal pyramidal cells (from 16 to 23) and ipsilateral corticocortical pyramidal cells (from 22 to 28); (Farinas and DeFelipe 1991). This not only suggests a preference for the innervation of callosal and ipsilateral corticocortical populations over cortico-thalamic projecting pyramidal neurons but also may indicate specific avoidance of the latter. This possibility is supported by the fact that other types of interneurons form occasional synapses with the AISs of pyramidal cells (Peters and Fairén 1978; Peters and Proskauer 1980; Somogyi et al. 1983; Kisvárday et al. 1985, 1987; DeFelipe and Fairén 1988; Gonchar et al. 2002), so the few axo-axonic synapses on cortico-thalamic projecting pyramidal neurons may not originate from ChCs. Whether this is true or is the case for other subpopulations of pyramidal cells in other cortical areas and species is unknown.

Furthermore, it has been shown that there are substantial differences in the distribution and density of GAT-1-ir Ch terminals in different areas and layers of the human and mouse neocortex. For example, the density of terminals innervating the AIS is not high in the primary sensory areas when compared to other areas like association areas in Human, and piriform and entorhinal cortex in mouse (Inda et al. 2007, 2009), Moreover, these differences were not correlated with the local neuronal density (Inda et al. 2007, 2009). Therefore, these differences might be related to the functional attributes of the cortical regions examined. ChCs are the major or sole source of synapses on pyramidal cell axon initial segments, and each cartridge innervates a single AIS. However, a single AIS may be innervated by one or few cartridges (five or less) which, in turn, may originate from the same or different ChCs (Fairen and Valverde 1980; Peters et al. 1982; Freund et al. 1983; reviewed in Somogyi et al. 1982, 1983; DeFelipe and Fariñas 1992).

Normal morphological development of the ChC axonal arbor and cartridges has recently been shown in the mouse to be regulated by DOCK7, a molecule member of the DOCK180 family, via the cytoplasmic activation of ErbB4 (Tai et al. 2014). Knockdown of either DOCK7 or ErbB4 at embryonic day 12.5 causes disorganization of the axonal tree and a decrease in the number and size of terminal boutons in mice sacrificed on postnatal day 28 (P28). Interestingly, when DOCK7 loss of function is induced in P7–P8 pups, it causes a decrease of bouton size and density while no apparent axonal phenotype is observed at P28. These findings suggest that the structure of the ChC axonal tree is established before the final maturation of terminal cartridges and, once established, it persists even if DOCK7 is no longer expressed. However, these data should be interpreted cautiously in the context of our present work since, although Tai et al. have indeed quantified bouton densities and sizes, their study on axonal structure was only qualitative and would benefit from a methodological approach such as the one we have developed here. Finally, this line of research is not only relevant to the examination of inhibitory cortical circuits in the normal brain, but also in brain diseases. Indeed, the deletion of ErbB4 in fast-spiking interneurons, which include Ch cells, has been shown to elicit a plethora of functional deficits that may be related to the pathophysiology of schizophrenia (Del Pino et al. 2013).

In summary, we conclude that the spatial positions of pyramidal cells innervated by a single ChC follow a clustered pattern so that single ChCs may exert a strong, widespread influence on their local neighbor pyramidal neurons in a spatially heterogeneous fashion. Such a clustered pattern of innervation strongly suggests the existence of target selectivity and/or avoidance, although it could also arise partly due to methodological limitations, or may reflect the stochastic peculiarities of axonal branching. The nature and possible functional significance of the clustered distribution of the cartridges as opposed to random or regular distributions should be further investigated.
